# Determination of the influence of variation in the number of layers in multilayer coatings on the inhibition of hydrogenation processes and thermal barrier characteristics

**DOI:** 10.1039/d5ra00926j

**Published:** 2025-07-04

**Authors:** G. Zh. Moldabayeva, A. L. Kozlovskiy, S. Zh. Abileva, M. A. Sadvakassov, Sh. R. Tuzelbayeva

**Affiliations:** a Department of Petroleum Engineering, Satbayev University Almaty 050013 Kazakhstan; b Laboratory of Solid State Physics, The Institute of Nuclear Physics Ibragimov st. 050032 Almaty Kazakhstan kozlovskiy.a@inp.kz

## Abstract

The evaluation results of the application of multilayer AlN–TiO_2_ coatings as protective thermal barriers and anticorrosive coatings that protect metal structures from corrosion processes during hydrogenation are presented. During the studies conducted, it was determined that increasing the number of layers in the coatings while maintaining the overall thickness allows for an increase in resistance to external mechanical effects (pressure and friction). This is because of the presence of interlayer boundaries that prevent the propagation of microcracks under external loads. At the same time, increasing the number of layers from 10× to 20× results in a slight decrease in adhesive strength due to a reduction in the adhesion of the layers with small thicknesses (approximately 50 nm). Simultaneously, the presence of a large number of layers inhibits hydrogen diffusion mechanisms in the coatings, which is expressed not only as an increase in resistance to cracking during indentation of coating samples after hydrogenation but also as less pronounced morphological changes in the topography of the coating surface associated with the formation of hillock-like gas-filled inclusions on the surface. An analysis of the thermal insulation characteristics of the coatings revealed that an increase in the number of layers leads to an increase in the temperature difference between the front and back sides, indicating an increase in thermal insulation due to the presence of interlayer boundaries, which leads to a slowdown in heat transfer.

## Abstract 2

The evaluation results of the application of multilayer AlN–TiO_2_ coatings as protective thermal barriers and anticorrosive coatings that protect metal structures from corrosion processes during hydrogenation are presented.

## Graphical abstract

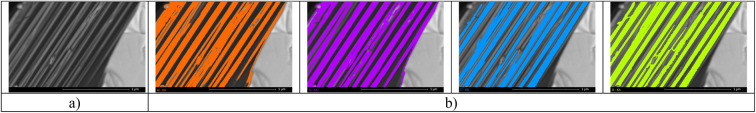

## Introduction

Currently, the use of protective coatings for metal structures is one of the most promising and economically viable methods for combating surface corrosion and the associated material degradation, which not only causes embrittlement but also increases the likelihood of critical situations.^1–3^ In most cases, corrosion processes that occur during operation are accompanied at the initial stage by the formation of pits and microcracks. The growth of these defects, driven by prolonged exposure of metal structures to aggressive environments, accelerates degradation processes.^4,5^ In addition to the corrosion processes associated with the formation of pits or microcracks, gas-filled inclusions may develop in the structure of the near-surface layer in several cases. These are often caused by the processes of hydrogen accumulation in pores, which at high concentrations can lead to the loss of strength and ductility as well as accelerated growth of microcracks.^6–8^ The mechanisms of hydrogen penetration into the near-surface layers include processes of electrochemical corrosion reactions, in which hydrogen is released in the form of by-products during penetration from hydrogen-saturated environments and long-term interaction of metal surfaces with environments containing high concentrations of hydrogen, typically observed under operating conditions. For example, in oil and gas main pipes, hydrogen cracking is one of the key factors causing emergency situations.^9,10^ Moreover, in contrast to the use of inhibitors or cathodic protection, the use of protective coatings makes it possible to increase the resistance of materials to degradation by inhibiting oxidation or hydrogenation processes (in cases where operating conditions are accompanied by exposure to gases or hydrogen sulfide solutions).^11,12^ The key mechanism underlying the creation and subsequent use of protective coatings is the creation of a barrier between the metal surface and the environment to reduce the contact of the metal with corrosive components, such as water, air, and aggressive environments, including acids, alkalis or petroleum products.^13–15^ The most promising method of application of such coatings is the magnetron sputtering method, the use of which not only makes it possible to control the possibility of applying coatings of a certain thickness with high precision, including on fairly complex multi-profile surfaces, but also creates multi-layer coatings with higher mechanical and anti-corrosion characteristics, including resistance to hydrogen accumulation processes or the formation of gas-filled inclusions.^16–19^ The use of multilayer coatings as protective materials, despite the fairly large prospects for practical application, requires significant efforts in the field of studying the possibility of combining various factors associated with both the composition of the coatings and their geometric dimensions (thickness, number of layers, grain sizes), which opens up fairly large prospects for conducting scientific research in this area.^19,20^

In recent times, an important role has been played by protective coatings, not only in protecting the surface of metal structures from corrosion but also as thermal barrier coatings that reduce metal overheating, leading to the acceleration of corrosion and destruction processes.^21,22^ In this case, the possibility of combining anti-corrosion and thermal insulation characteristics in protective coatings allows them to be used in various extreme conditions, which in turn expands the horizons of their practical application.

The main aim of this study is to investigate the prospects for using oxy-nitride coatings as thermal barrier and anti-corrosion protective coatings that can reduce the destructive destruction of near-surface layers of steels used in extreme conditions. The choice of composite multilayer oxy-nitride coatings obtained by alternating layers of aluminum nitride and titanium dioxide as objects of study is due to the possibility of combining high hardness, adhesive strength, anti-corrosion and thermal insulation characteristics. At the same time, emphasis is placed on the influence of the number of layers and their thickness on the effectiveness of protection against hydrogenation and subsequent destruction.

## Experimental part

**Fig. 1:**
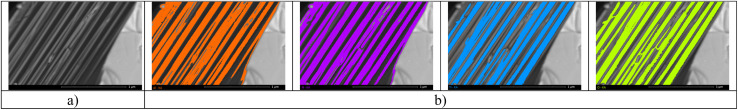
(a) SEM image of the side cleavage of the coating; (b) results of mapping of the side cleavage of the coating.

The samples used in this study were oxy-nitride coatings obtained by magnetron sputtering by layer-by-layer deposition of aluminum nitride (AlN) and titanium dioxide (TiO_2_). Aluminum nitride and titanium oxide targets purchased from the company (K. Lesker, USA) were used as deposition targets. Layers were applied layer by layer by changing the sputtering modes and targets. The following sputtering conditions were selected for the application of aluminum nitride: the discharge power was 250 W, the working gas was a mixture of argon and nitrogen in a ratio of 40/60, and the gas supply pressure was 5 × 10^−3^ bar. For the application of titanium oxide, the working gas was a mixture of argon and oxygen in a ratio of 45/55, the gas pressure was 6 × 10^−3^ bar, and the discharge power was 250 W. The use of these gas mixtures and the selected sputtering conditions for each sputtering type eliminates the formation of impurity inclusions in the resulting coating composition. The thickness control was performed using the ellipsometry method. The analysis of the layers, performed using the elemental analysis method presented in ref. 23, showed the absence of inclusions in the composition of the layers during their application and the uniformity of sputtering under the specified operating conditions. According to the elemental composition data, in the case of aluminum nitride layers, the elemental ratio Al : N is 47.8% : 52.2%, which is close to the stoichiometric ratio of elements characteristic of the AlN phase. In the case of TiO_2_, the Ti : O elemental ratio is approximately 31.3% : 68.7%, which has a slight deviation from the stoichiometry of the TiO_2_ phase. Fig. 1 shows an image of a side cleavage of the obtained coating, reflecting the layered structure of the obtained coatings, and the presented mapping results show the absence of layer mixing effects during spraying.

The total thickness of the samples studied was about 1 μm, while varying the number of layers led to a decrease in the layer thickness in order to maintain the total thickness of the sample. Therefore, samples with layer thicknesses of approximately 250, 200, 100 and 50 nm were obtained, which made it possible to obtain 4-, 5-, 10- and 20-layer coatings with alternating layers. Regarding the use of the term oxynitride coatings, in this context, this term is used to reflect the fact of using alternating layers of titanium oxide and aluminum nitride by applying them using the magnetron sputtering method. The formation of oxynitride phases during the sputtering process is not possible because sputtering occurs under different conditions; the boundary of the layers during their sputtering and partial mixing is the only place where such phases can form. However, recording such phenomena is extremely difficult for the following reasons. The use of the standard X-ray diffraction method does not allow recording well-structured phases in the coatings due to the small thickness of the layers. In this case, the X-ray diffraction patterns are characterized by the presence of a halo, which can be associated with the small size of the particles from which the layers are formed and the formation of amorphous coatings due to the small thicknesses. The coating samples were applied to the surface of X80 steel, used as a reference for conducting hydrogenation and corrosion experiments, as well as for evaluating the thermal insulation parameters. Previously, in ref. 23 and 24, a method for applying similar coatings to 316L steel was demonstrated, and the efficiency of using multilayer coatings for protection against destruction during corrosion and wear was determined.

The strength characteristics were studied by measuring the hardness and adhesive strength depending on the variation in the number of layers in the coatings. The indentation method was used to measure hardness using a Duroline M1 microhardness tester (Metkon, Bursa, Turkey). The measurements were carried out in the low-load mode (the indenter load was 10 N), which made it possible to determine the coating hardness during indentation. The adhesive strength was determined using the surface scratching method implemented on the Unitest framework SKU UT-750 testing machine (Unitest, USA).

Visualization of the structural features of the surface was performed using the atomic force microscopy method implemented using a Smart SPM microscope (AIST-NT, Zelenograd, Russia). The surface topography was recorded in a semi-contact mode with the ability to visualize the structural effects associated with the formation of gas-filled bubbles on the surface during hydrogen accumulation, as well as changes in the surface roughness as a result of degradation during hydrogenation.

**Fig. 2:**
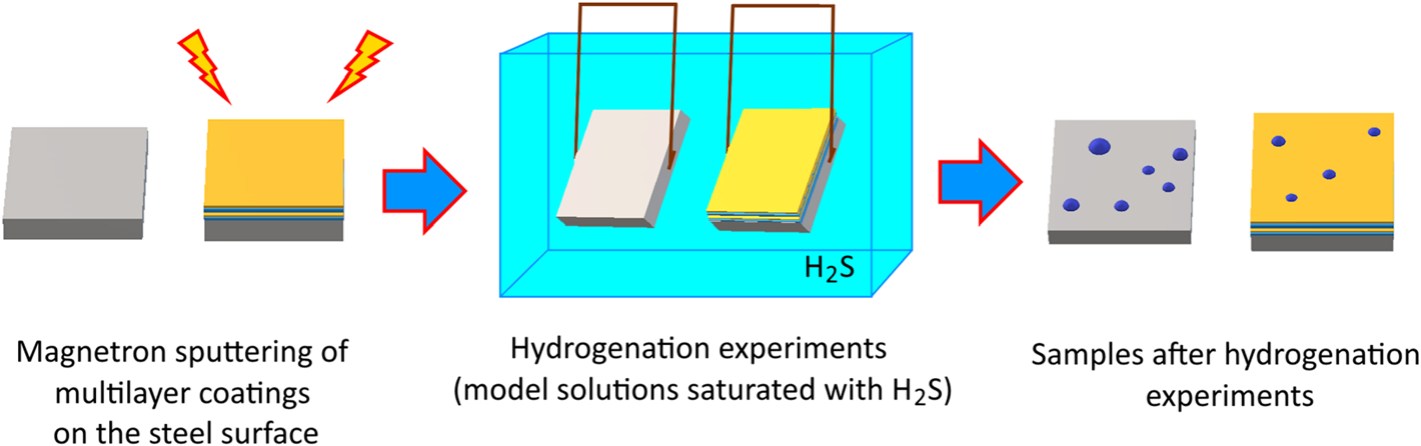
Scheme of experiments on the hydrogenation of samples.

To determine the degradation kinetics associated with hydrogen accumulation processes and subsequent hydrogen embrittlement and cracking, hydrogenation experiments were performed on specimens according to a standard procedure corresponding to NACE TM0284. Special chamber boxes were used for testing, in which the samples were placed in a suspended state. In this case, in order to exclude the initiation of destruction processes from the reverse side, the reverse side of the samples was isolated from contact with the environment of the model solution. Fig. 2 shows the basic scheme for obtaining coatings with subsequent hydrogenation experiments. The experiments consisted of placing the studied samples with and without applied coatings in model solutions saturated with H_2_S and holding them in the solutions for 10 days. After every 24 hours (1 day), measurements of adhesive strength and hardness were carried out. The hydrogenation experiments were conducted in strict accordance with NACE TM0177-2024 (SSC, HIC, SCC) and ISO 15156-1:2020 (MR0175) protocols and were conducted in a fume hood using a dual scrubber and a gas analyzer to monitor the conditions inside the hood throughout the experiment. The H_2_S concentration in the model solution is maintained by continuously feeding gas into the solution in order to avoid changes in the H_2_S concentration in the model solution, in order to eliminate the effect of uneven H_2_S exposure over time. The gas was fed through a glass diffuser using the continuous fine-bubble bubbling method at a rate of 0.5 mL min^−1^, and the pressure was controlled using special setters-regulators. After 10 days, wear resistance and thermal insulation tests were conducted, which made it possible to evaluate the change in resistance to surface degradation and the effect of hydrogenation processes on thermal insulation properties.

The determination of tribological characteristics associated with surface wear due to hydrogenation and destruction during surface corrosion, as well as the assessment of the effect of using protective coatings on resistance to surface degradation and wear, was carried out by measuring the dry friction coefficient of the samples under study. A Nanovea T100 tribometer (Nanovea, Irvine, CA, USA) was used to perform the tests.

The measurements were performed using the “ball on disk” scheme with a load of 50 N on the ball, with a 5 mm diameter aluminum oxide ball used as a counterbody. A comparative analysis of the values of the dry friction coefficient before and after hydrogenation tests made it possible to determine not only the kinetics of surface degradation but also the effectiveness of using protective coatings to increase corrosion resistance.

The thermal insulation assessment was carried out by conducting tests related to measuring the temperature difference between the front and back sides of the steel samples under study, with and without protective coatings. The samples were heated to 200 °C for 24 hours, and measurements were taken using thermocouples placed on the front and back sides. Based on the data obtained, the temperature difference (Δ*T*) was measured, the value of which determines the ability of the coating to withstand the negative impact of temperature.

## Results and discussion

**Fig. 3:**
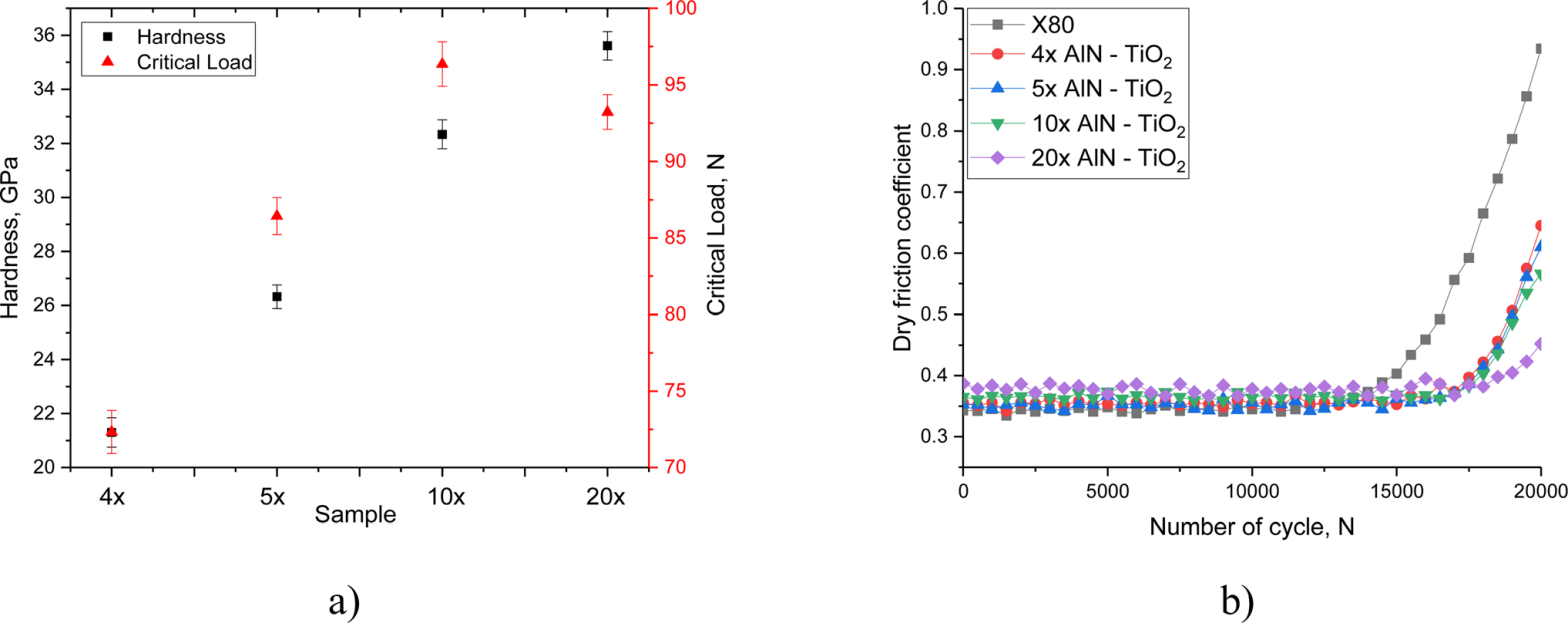
(a) Assessment results of strength characteristics (hardness and adhesive strength) depending on the number of layers in the samples; (b) results of tribological tests of the studied coating samples based on the number of layers in alternation.

Fig. 3 shows the results of the evaluation of the strength (hardness and adhesion strength) and tribological (dry friction coefficient) characteristics of the coatings under study, depending on the number of layers during spraying. The results of the tribological tests are presented together with the data obtained for X80 steel, in order to compare the wear resistance of the surface. The general appearance of the presented data on the change in the hardness of the studied coating samples depending on the number of layers indicates a positive effect of increasing the number of layers during spraying on the resistance to external mechanical impacts exerted by the indenter when a constant load is applied. An increase in the number of layers in coating samples leads to an increase in hardness, which is due to the effect of an increase in the number of interlayer boundaries, the presence of which leads to an increase in resistance to cracking under external loads, as well as an increase in resistance to plastic deformation under external static pressure on the surface. The observed strengthening mechanism in this case is due to the presence of interlayer boundaries that prevent the initiation and propagation of microcracks under pressure. At the same time, increasing the number of layers from 4 to 20 increases the hardness from 21 GPa to 36 GPa, which is more than 60% of the increase in strengthening efficiency caused by a change in the number of layers. However, when assessing the adhesive strength, reducing the size of the layers from 100 to 50 nm leads to a decrease in the adhesive strength, *i.e.* adhesion to the steel surface, which is expressed in a decrease in the critical load from 96 N to 93 N, necessary to tear the coating off the surface. In this case, thinner layers under external peel load reduce stability and adhesion to the surface, resulting in a decrease in the strength of the peel. However, compared with the 4-layer and 5-layer coatings, the adhesive strength value for the 20-layer coatings is significantly higher. The observed strengthening effects due to a change in the number of layers in the coatings are in good agreement with the results of works,^25,26^ in which the observed strengthening effects are associated with the Hall–Petch model, which is based on the containment of dislocations at the boundaries of layers, leading to strengthening and an increase in cracking resistance. In this case, a decrease in the thickness of the sprayed layers, with their alternation and an increase in the number of layers, leads to the creation of barriers for dislocations, which leads to their containment at the boundaries, resulting in an increase in resistance to deformation. In this case, the observed strengthening effect can be explained by the fact that when the layer thickness is reduced by reducing the spraying time, the grains formed have much smaller sizes than with thicker layers, which also leads to the formation of the effect of dispersion strengthening, caused by the presence of grain boundaries in the layer, preventing the spread of deformation-induced cracks during indentation,^27^ as well as their spread when the load changes. However, in this case, the decrease in resistance to separation observed for samples of 20-layer coatings can be explained by the fact that the containment of dislocations in small volumes of layers leads to the creation of locally deformed regions in the structure, which, under mechanical translational loading, lead to the separation of layers from each other in places where the concentration of these inclusions is high.

The results of the dry friction coefficient assessment presented in Fig. 1b show the effectiveness of increasing the wear resistance of the surface in the case of using multilayer coatings in comparison with the results of changes obtained for X80 steel used as a substrate onto which the coatings were applied. In this case, the most pronounced changes in the dry friction coefficient were observed for samples obtained by alternating 20 layers of aluminum nitride and titanium dioxide, for which the value of the dry friction coefficient after 20 000 test cycles was 0.45 (with an initial value of 0.39), which is more than two times less than the value of the dry friction coefficient obtained after 20 000 cycles when testing X80 steel. The increase in wear resistance of coatings in this case can also be explained by the effects associated with a change in the number of layers and, as a direct consequence, a change in the grain size, dislocation density and interlayer boundaries that prevent plastic deformation under external influences. The complication of the microstructural features of coatings with an increase in the number of layers and a decrease in their thickness leads to an increase in resistance to the propagation of microcracks by inhibiting their development in the zones of interlayer boundaries, as well as the retention of dislocations at grain boundaries, preventing their agglomeration.^28,29^

One of the key areas of applicability of coatings, including multilayer coatings, is the creation of a barrier layer that prevents or slows down the corrosion and degradation processes of steel during hydrogenation, processes in which the introduction and subsequent accumulation of hydrogen in the near-surface layers occurs, which can cause deformation distortions of the structure. By its nature, hydrogen quite easily penetrates into the near-surface layers of metals and agglomerates in micropores or near interphase boundaries, leading to the formation of gas-filled inclusions, the increase in volume of which in turn leads to brittle fracture and cracking. At the same time, prolonged contact of metal surfaces with aggressive environments, including H_2_S, can lead not only to the penetration of a high concentration of hydrogen into the near-surface layers but also accelerate oxidation processes due to deformation swelling, leading to an increase in the size of microcracks and pits, thereby weakening the strength parameters. Consequently, a double threat associated with the initiation and acceleration of classical corrosion processes due to oxidation may arise when interacting with aggressive environments. In this case, the degradation processes can be intensified by hydrogen penetration and agglomeration in the pores and microcracks formed due to corrosion. At the same time, the use of coatings makes it possible to reduce the rate of hydrogen penetration, and structural features associated with the presence of interlayer boundaries and small grain sizes make it possible to create additional barriers to contain the diffusion of hydrogen into the metal, thereby slowing down the processes of destruction during hydrogenation and long-term interaction with aggressive environments. Such coatings act as so-called “sacrificial” coatings, the application of which implies containing and slowing down corrosion processes with the greatest possible efficiency.

**Fig. 4:**
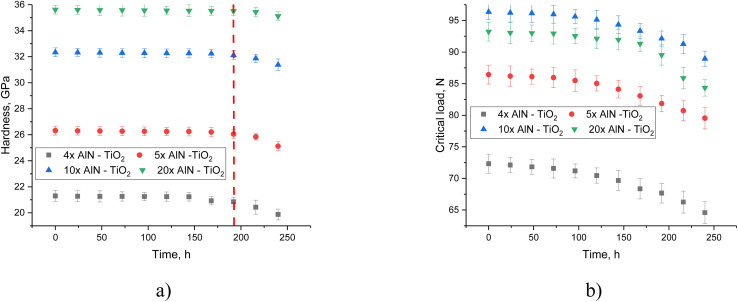
Results of the assessment of changes in the strength characteristics of the studied coatings subjected to surface hydrogenation: (a) results of changes in the hardness of the coatings during hydrogenation experiments; (b) results of the assessment of changes in the value of adhesive strength.

Fig. 4 shows the assessment results of the change in the strength characteristics of the coatings under study after hydrogenation, reflecting the degradation of the surface associated with the accumulation of hydrogen in the damaged coating layer, leading to destabilization due to deformation distortion of the structure. The most significant changes in hardness values occurred after holding in the model solution for more than 180 hours, while the trend of hardness change varied with the type of coating, indicating a direct effect of the number of layers in the coating on resistance to hydrogen embrittlement. Analysis of changes in hardness values after 240 hours of testing revealed that in the case of 4- and 5-layer coatings, it is approximately 7% and 5%, respectively, while for 10- and 20-layer coatings, the decrease in hardness after 240 hours is less than 2–3%. Such differences indicate that an increase in the number of layers, with a decrease in their thickness, leads to the formation of barrier boundaries that reduce the diffusion of hydrogen deep into the material, thereby slowing down the destruction process and reducing resistance to external influences.

In the case of changes in the value of the adhesive strength for peeling off the coating depending on the hydrogenation time, the most significant changes were observed after 100 hours of hydrogenation, upon which a clearly expressed trend of decreasing the values of the critical load necessary for peeling off the coating from the steel surface was observed. The absence of significant changes in the value of the critical load during testing up to 100 hours indicates that the main changes in the adhesive strength are primarily associated with the accumulation of hydrogen and its agglomeration in the near-surface layer, which leads to a weakening of the adhesion of the coating to the steel surface. In this case, the growth of gas-filled inclusions during hydrogen accumulation leads to an increase in deformation distortions that have a negative effect on the coatings during their growth, which leads to the destabilization of the crystalline structure, as well as the partial destruction of interphase and chemical bonds, the weakening of which leads to a loss of strength.

**Fig. 5:**
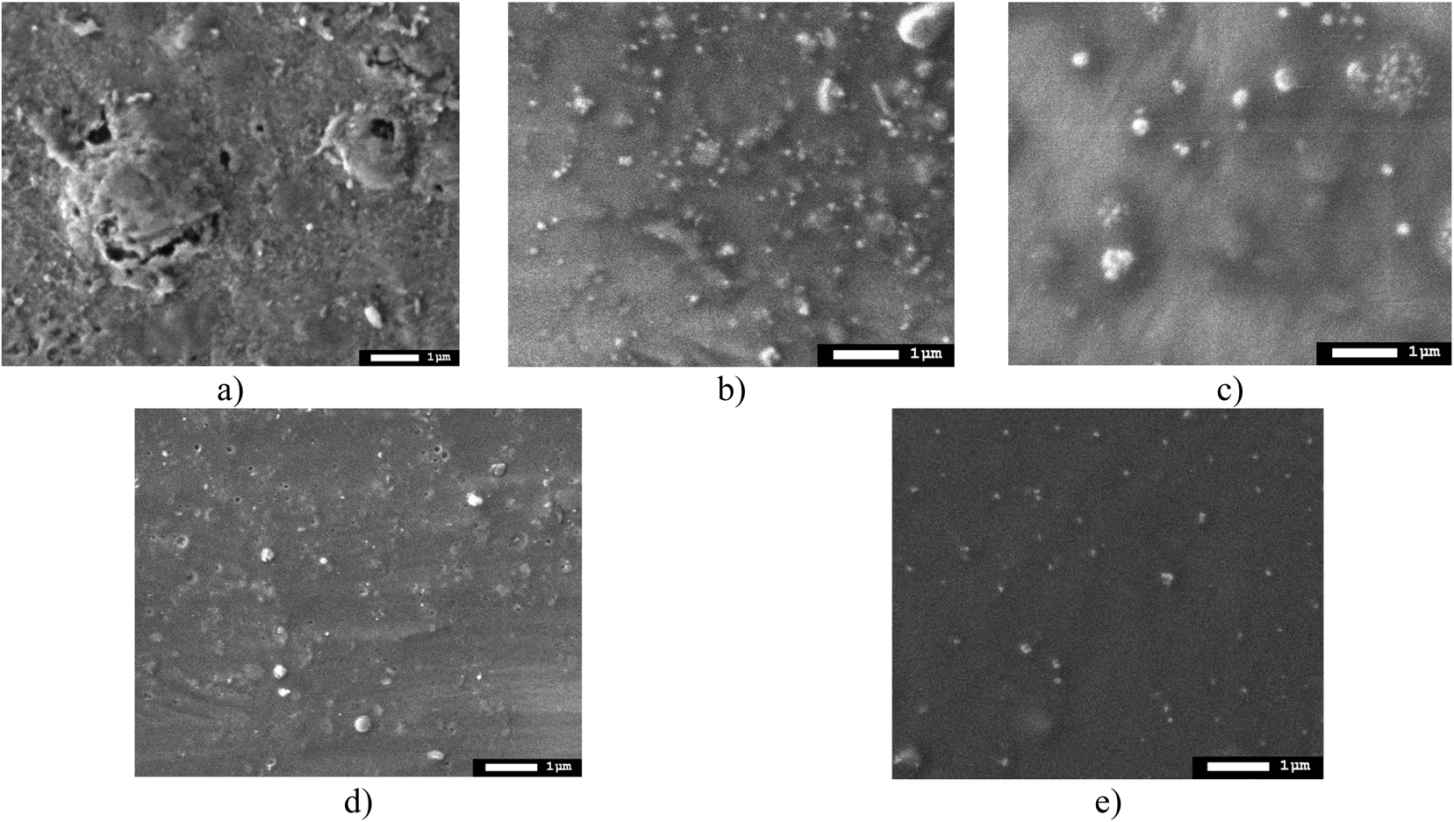
Results of morphological studies of samples after hydrogenation, performed using the scanning electron microscopy method: (a) X80 steel; (b) 4× AlN–TiO_2_ films; (c) 5× AlN–TiO_2_ films; (d) 10× AlN–TiO_2_ films; (e) 20× AlN–TiO_2_ films.

Fig. 5 shows SEM images of the surface of the X80 steel samples under study, as well as coatings applied to the steel surface after hydrogenation tests, which demonstrate the surface degradation processes. In the case of X80 steel, bulges and cavities are visible on the surface, the presence of which indicates the destruction of the near-surface layer during hydrogenation of the surface. Moreover, in the case of applied coatings, a decrease in the surface degradation degree is observed according to the SEM images, and in the case of samples with 10 and 20 layers, the main changes are associated with the formation of small spherical inclusions, indicating the process of formation of gas-filled pores. Such changes in this case indicate the inhibition of swelling mechanisms due to the presence of interlayer boundaries that prevent hydrogen migration and gas-filled inclusion formation.

**Fig. 6:**
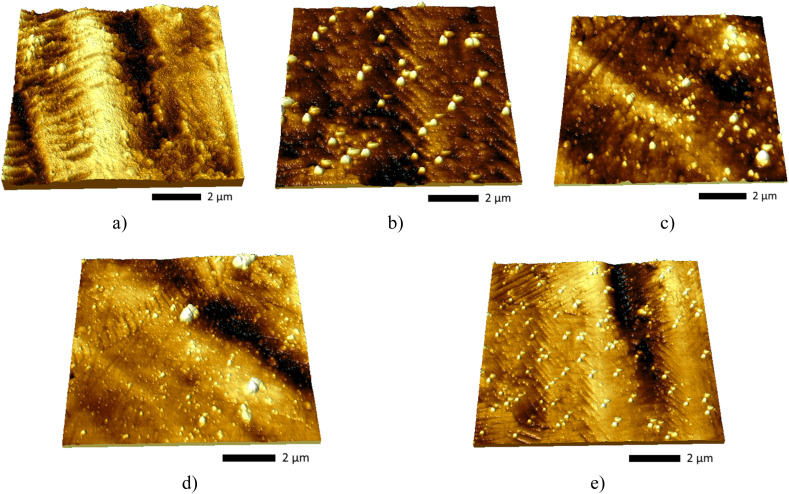
Results of 3D visualization of the surface of the studied samples after hydrogenation (color gradation from dark to light denotes a difference in the heights of structural formations formed as a result of hydrogenation): (a) X80 steel; (b) 4× AlN–TiO_2_ films; (c) 5× AlN–TiO_2_ films; (d) 10× AlN–TiO_2_ films; (e) 20× AlN–TiO_2_ films.

Fig. 6 shows the results of morphological studies reflecting the degradation of the surface due to hydrogenation in the case of X80 steel and coatings with different numbers of alternating layers. The data are presented in the form of a 3D visualization of the surface of the sample obtained by applying the semi-contact method of surface topography survey using atomic force microscopy. The presented data of changes in the surface relief of steel after hydrogenation show the formation of longitudinal waves with clearly expressed ridges and agglomerates of spherical inclusions, the presence of which is due to hydrogenation processes, manifested by the formation of gas-filled inclusions. The appearance of these inclusions on the surface was also established for samples with applied coatings after hydrogenation; however, the analysis of 3D images of the surface of samples with coatings indicates the influence of the variation in the number of layers on the effects of the formation of gas-filled inclusions in the structure. According to the presented data, an increase in the number of layers leads not only to a decrease in the size of these inclusions, which is seen when comparing the coatings obtained with 4-layer spraying and in the case of 10–20-layer spraying. In this case, an increase in the number of layers leads to a decrease in the volumetric dimensions of the gas-filled inclusions formed on the surface, the extrusion of which occurs at high concentrations due to the agglomeration of hydrogen in structural voids and pores. The observed inhibition of the growth of the volumetric dimensions of gas-filled inclusions in coatings due to a change in the number of layers can be explained by the presence of interlayer boundaries that prevent hydrogen diffusion in the layers, which leads to a slowdown in the growth of agglomerations, which in turn leads to the formation of smaller inclusions extruded onto the surface. It should also be noted that there is an observed heterogeneity in the volumetric sizes of grains, which manifests itself when the number of layers in coatings changes from 4 to 5 and 10 layers, especially for 10-layer coatings, in which the formation of fairly large agglomerates surrounded by small-sized inclusions several times smaller than large agglomerates is observed. By analyzing the obtained data, we can conclude that the use of multilayer coatings makes it possible to significantly slow down the diffusion of hydrogen in the near-surface layers, which reduces the effect of hydrogen cracking and embrittlement of the near-surface layers of metal structures. In this case, the slowdown in hydrogen diffusion is due to an increase in interlayer boundaries and layer sizes, which leads to a denser packing that prevents the growth of volumetric sizes of gas-filled voids during agglomeration and inhibits hydrogen penetration into the depths. In turn, the difference in the volumetric sizes of gas-filled inclusions for samples of 5×- and 10×-layer coatings can be explained by the presence of weakly bound grains, which leads to more pronounced deformation during hydrogen accumulation and accelerated growth of these inclusions. In turn, an increase in the number of alternating layers makes it possible to reduce this effect, which leads to the formation of a large number of small gas-filled inclusions, the presence of which results in less pronounced deformation and degradation of the surface layer. A similar effect of restraining the swelling of gas-filled agglomerates in the structure of the damaged layer was observed in the case of using multilayer coatings to protect against radiation-induced swelling.^30–32^ According to the presented results of the work, changing the number of layers during their alternation leads to the inhibition of the mechanisms of gas swelling associated with the agglomeration of implanted ions in the near-surface layers, which leads not only to a slowdown in the processes of destruction at high concentrations of implanted ions but also to a significant shift in the critical doses of damage at which destructive embrittlement and cracking are observed.

In this case, analyzing the data obtained using scanning electron microscopy, as well as the provided 3D images of the surface, which reflect changes in the surface caused by hydrogenation processes, it can be concluded that an increase in the number of layers in the coating samples leads to a significant inhibition of the processes of surface swelling during hydrogenation. Conclusions about the inhibition of the formation of the effect of resistance to oxidation processes are made considering the structural features of the coatings associated with the number of layers in the coatings, which, as the results of the studies show, play a key role in the inhibition of the destruction processes.

**Fig. 7:**
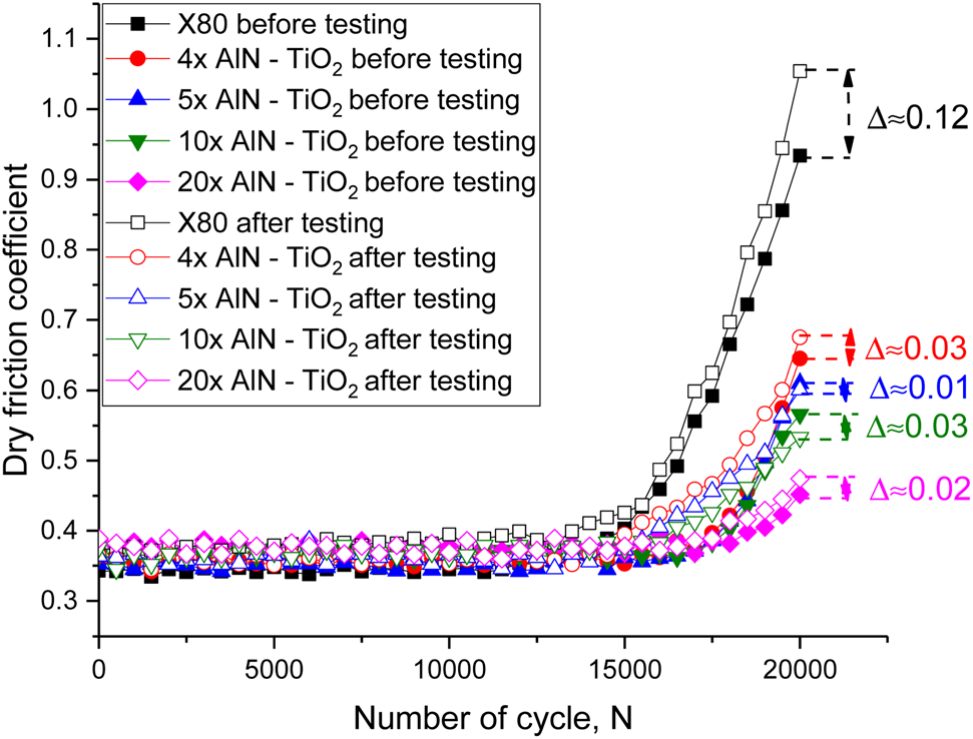
Results of tribological tests of the surface of the coating samples and X80 steel before and after the hydrogenation tests, reflecting the kinetics of surface degradation due to the deterioration of wear resistance parameters caused by the accumulation of structural defects during the formation of gas-filled inclusions (the *Δ* value reflects the difference between the maximum values of the friction coefficient, indicating the coefficient degradation degree during hydrogenation).

Fig. 7 demonstrates the assessment results of the dry friction coefficient of the studied samples in comparison before and after tests associated with hydrogenation for 24 hours, which characterizes the degradation of the wear resistance of the surface caused by the accumulation of deformation distortions caused by the formation of gas-filled inclusions in the near-surface layer. The general appearance of the observed changes in the value of the dry friction coefficient for samples after hydrogenation indicates the most pronounced degradation of the surface of X80 steel, for which the difference in the dry friction coefficient before and after hydrogenation upon reaching 20 000 cycles is approximately 0.12, which is 4–6 times greater than the similar difference in the coefficients obtained in tribological tests of coatings. In this case, such a strong difference in the dry friction coefficients for the samples is due to differences in surface degradation observed in the 3D images shown in Fig. 3. According to the visualization results obtained in the case of X80 steel, pronounced surface degradation causes more pronounced destruction and loss of mass during wear, leading to more pronounced changes in the dry friction coefficient. At the same time, the shift of the initial stage of changes in the coefficient of dry friction from the number of cycles for testing samples subjected to hydrogenation indicates a decrease in the resistance of the surface to wear due to the destruction of the near-surface layer caused by the accumulation of gas-filled inclusions.

**Fig. 8:**
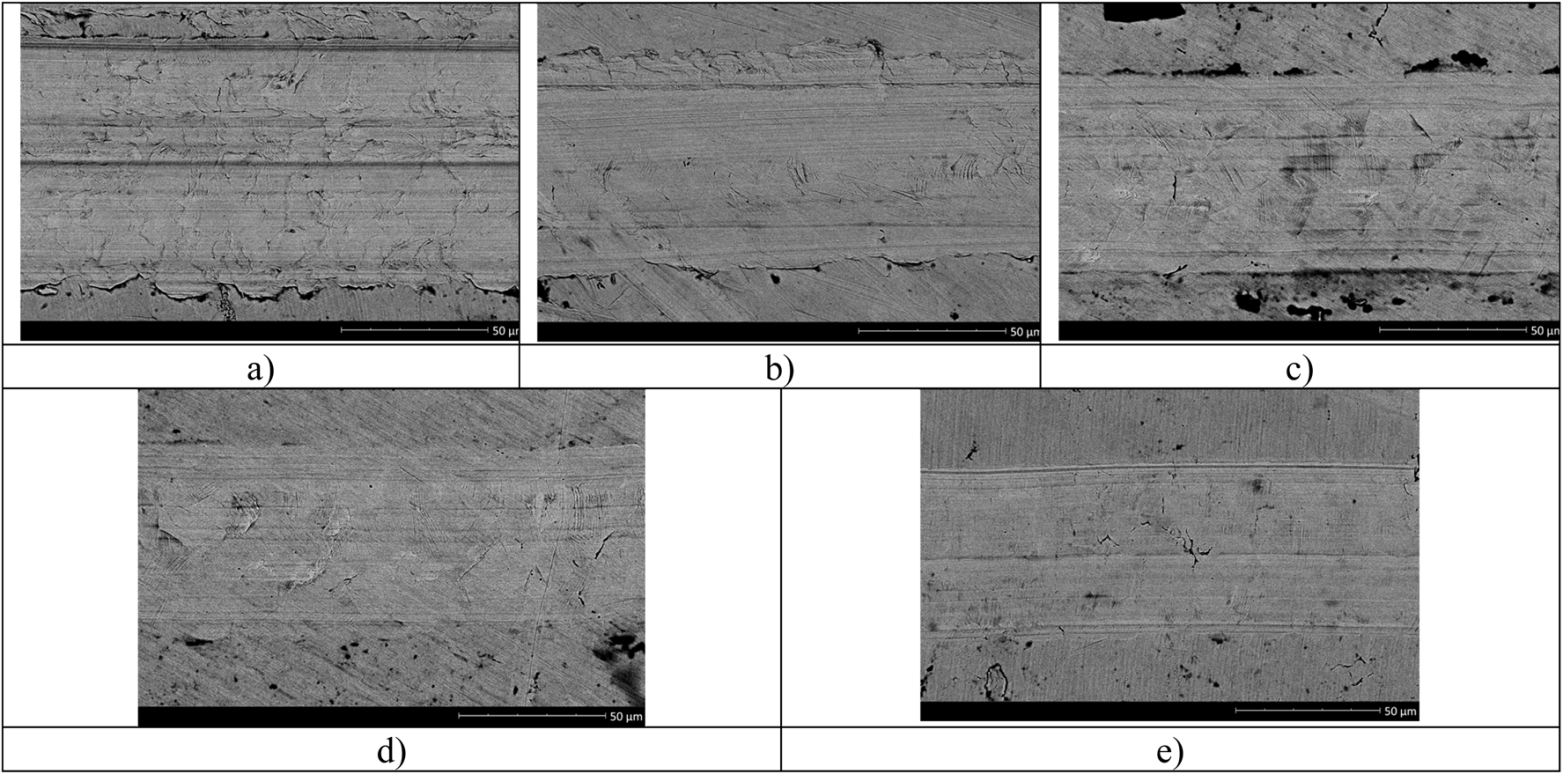
SEM images of surface wear profiles after tribological testing: (a) X80 steel; (b) 4× AlN–TiO_2_ films; (c) 5× AlN–TiO_2_ films; (d) 10× AlN–TiO_2_ films; (e) 20× AlN–TiO_2_ films.

Fig. 8 shows wear profile images after tribological tests, reflecting the effect of varying the number of layers on the wear resistance of coatings. As shown in the images, an increase in the number of coating layers leads to a decrease in surface wear, which is a direct confirmation of the results presented in Fig. 7.

**Fig. 9:**
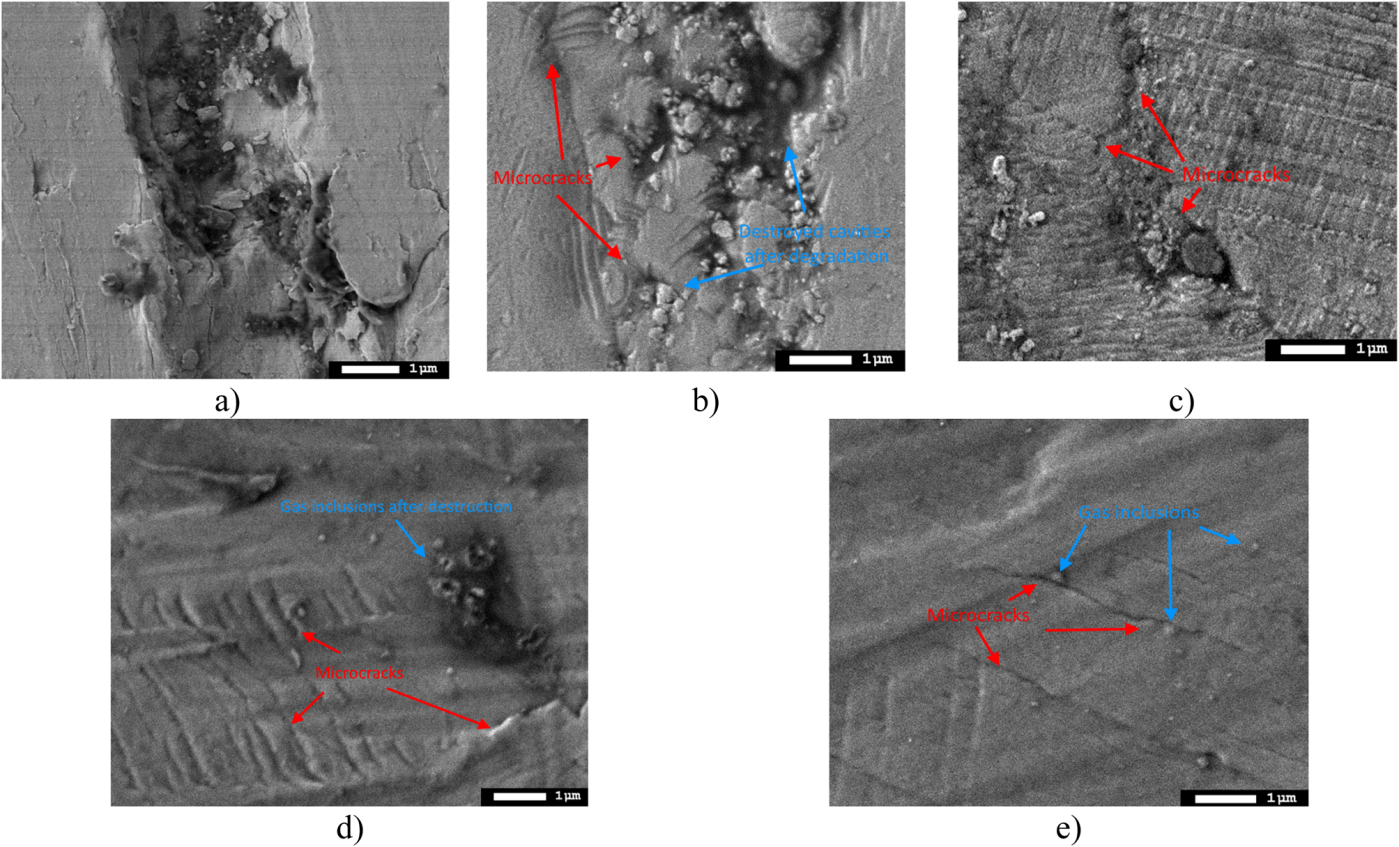
Results of morphological features of the surface of the studied samples subjected to hydrogenation after tribological tests: (a) X80 steel; (b) 4× AlN–TiO_2_ films; (c) 5× AlN–TiO_2_ films; (d) 10× AlN–TiO_2_ films; (e) 20× AlN–TiO_2_ films.

Fig. 9 shows the surface morphology assessment results of the samples subjected to hydrogenation after the wear resistance tests. The obtained surface images reflect the effects of hydrogenation on surface degradation, leading to increased damage, microcrack formation and partial opening of gas-filled bubbles on the surface.

**Fig. 10:**
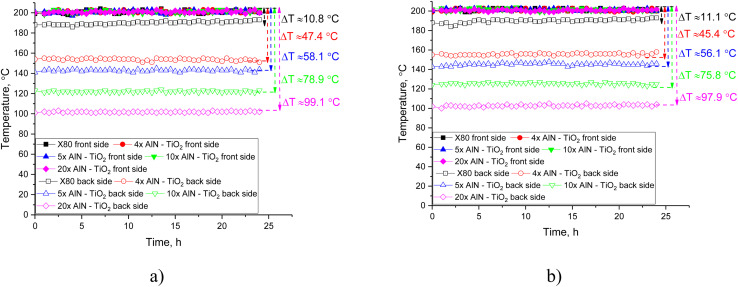
Test results of samples for determining the thermal insulation properties of the coatings, expressed as dependencies of temperature changes on the front and back sides (Δ*T* reflects the difference in temperatures on the front and back sides, showing the effectiveness of heating processes): (a) results of temperature alteration of the samples depending on the number of applied layers; (b) results of temperature changes on the front and back sides of the samples after hydrogenation tests.

Fig. 10 shows the thermal insulation test results of the investigated coating samples in the initial state and after the hydrogenation tests. The data are presented as dependencies of temperature changes on the front and back sides when heating one of the sides, and the magnitude of the difference shows how much the heating processes are restrained by the applied coating. The data are also shown for X80 steel, which was used as a comparison sample in this experiment. Based on the temperature difference between the front and back sides, a thermal insulation effect was established, reflecting the efficiency of using protective coatings against overheating, which can lead to destabilization and acceleration of corrosion processes of the metal surface. In the case of X80 steel without applied coatings, the difference Δ*T* is about 10–11 °C, indicating low heat loss and fairly good thermal conductivity of steel, which leads to almost uniform heating from both sides (the difference in Δ*T* is less than 10% of the initial heating temperature).

As shown in the presented data, the formation of protective coatings on the surface of X80 steel with a different number of applied layers with their alternation allows elevation of the difference in Δ*T* from 47 °C to 99 °C, which is from 25% to 49% of the initial heating temperature of the front side. The observed effect of thermal insulation, as well as changes in the value of Δ*T* depending on the number of applied alternating layers in this case, can be explained by the effects of the presence of interlayer boundaries, as well as dimensional factors that limit (restrain) phonon heat dissipation in samples during heat transfer, which leads to a decrease in thermal conductivity and an increase in thermal insulation. This effect allows the temperature effect to be reduced during operation under high-temperature conditions, eliminating the effects of heating. Thus, the use of protective coatings that prevent wear during hydrogenation (see the data of experiments on hydrogenation) also makes it possible to consider them as heat-insulating coatings. At the same time, the results of tests on the heat insulation of X80 steel samples and coatings after hydrogenation for 240 hours, presented in Fig. 5b, also indicate the preservation of the stability of heat insulation during hydrogenation. The observed difference in values, which is approximately 1–2 °C, can be explained by structural defects caused by destruction processes during hydrogenation, associated with the formation of gas-filled inclusions, leading to destabilization.

Summarizing the overall results of the measurements of thermal insulation properties, it can be concluded that an increase in the number of layers and the difference in the thermal expansion coefficients of titanium dioxide and aluminum nitride have a positive effect on the thermal insulation properties of the coatings. An increase in the number of layers leads to the creation of additional barrier effects that restrain phonon heat transfer due to rescattering, which leads to a slowdown in heat transfer and increases the temperature difference between the front and back sides.

Table 1 shows the generalized assessment results of changes in the strength and thermal insulation properties of coatings, depending on the number of layers during spraying, allowing the evaluation of the most promising compositions and manufacturing conditions.

**Table 1:** Assessment results of changes in the strength and thermal insulation properties of coatings

Sample	Strength parameters				
	Strengthening^a^, %	Increase in separation resistance, %	Thermal insulation efficiency^b^	Degree of surface wear compared with steel X80^c^	Degree of surface wear compared with steel X80 after hydrogenation
Steel X80	—	—	0	2.95 × 10^−5^	3.39 × 10^−5^
4× AlN–TiO_2_ films	0	0	4.3 times	1.45 × 10^−5^	1.55 × 10^−5^
5× AlN–TiO_2_ films	23.5	19.5	5.4 times	1.28 × 10^−5^	1.19 × 10^−5^
10× AlN–TiO_2_ films	51.8	33.2	7.2 times	1.01 × 10^−5^	0.78 × 10^−5^
20× AlN–TiO_2_ films	67.2	28.9	9.1 times	0.33 × 10^−5^	0.42 × 10^−5^

a The calculation was performed for samples in comparison with the 4×-layer coatings of AlN–TiO_2_.

b The calculation was performed by comparing the temperature difference values obtained for coatings and steel X80.

c The calculation was performed by calculating the difference in dry friction coefficients before and after 20 000 sequential tests, considering the total number of cycles.

As shown in the data presented, an increase in the number of layers from 4–5 to 10–20 makes it possible to increase the mechanical properties and also significantly increase the effect of thermal insulation, which positively affects the possibility of enhancement of the efficiency of operation in extreme conditions associated with high temperatures and operation in aggressive media. At the same time, the application of protective coatings makes it possible to significantly (multiply) enhance the wear resistance of the surface by increasing the resistance to friction and destruction associated with hydrogenation.

## Conclusion

According to the conducted studies, an increase in the number of layers in oxy-nitride coatings leads to an increase in the resistance to hydrogen absorption and corrosion processes associated with the accumulation of hydrogen in the near-surface layers. The increase in the number of layers from 10 to 20 at layer thickness reduction leads to a growth in hardness under static mechanical loads, whereas layer thickness reduction leads to a slight decrease in adhesive strength due to a decrease in adhesion between layers with their small thickness and a large number of alternations. According to the results of the tribological tests, the application of coatings leads to an increase in the wear resistance of the surface, as well as an increase in the resistance to hydrogenation and destruction due to surface corrosion. An analysis of the morphological features of the studied coatings showed that the use of multilayer coatings decreases hydrogen agglomeration, which is expressed in a decrease in the size of gas-filled inclusions on the surface. Therefore, it is promising to use 10–20 layer coatings to restrain hydrogen degradation and cracking in the event of surface hydrogenation during operation. The observed preservation of the stability of the thermal insulation properties of the coatings during hydrogenation indicates a small influence of the processes of hydrogen accumulation in the near-surface layer of the coating on the change in the heat exchange mechanisms. In this case, the accumulation of structural damage caused by hydrogenation leads to an increase in the dispersion of thermal phonons, which leads to an increase in heat losses.

## Author contributions

Conceptualization, G. Zh. M., A. L. K., M. A. S., S. Zh. A., Sh. R. T.; methodology, G. Zh. M., A. L. K., M. A. S., S. Zh. A., Sh. R. T.; formal analysis, G. Zh. M., A. L. K., M. A. S., S. Zh. A., Sh. R. T.; investigation, G. Zh. M., A. L. K., M. A. S., S. Zh. A., Sh. R. T.; resources, G. Zh. M., A. L. K., M. A. S., S. Zh. A., Sh. R. T.; writing—original draft preparation, review, and editing, G. Zh. M., A. L. K., M. A. S., S. Zh. A., Sh. R. T.; visualization, G. Zh. M., A. L. K., M. A. S., S. Zh. A., Sh. R. T.; supervision, G. Zh. M. All authors have read and agreed to the published version of the manuscript.

## Conflicts of interest

The authors declare no conflicts of interest.

## Data Availability

All data presented in the article were obtained using certified equipment and proven analysis methods. All experimentally obtained data are presented in the article, and additional data are available upon request. SEM images were obtained using a Phenom™ ProX scanning electron microscope (Thermo Fisher Scientific, Eindhoven, The Netherlands). Hardness was measured using the indentation method using a Duroline M1 microhardness tester (Metkon, Bursa, Turkey). The measurements were performed in the low-load mode (the indenter load was 10 N), which made it possible to determine the coating hardness during indentation. The adhesive strength was determined using the surface scratching method implemented on the Unitest framework SKU UT-750 testing machine (Unitest, USA). Visualization of the structural features of the surface was performed using the atomic force microscopy method implemented using a Smart SPM microscope (AIST-NT, Zelenograd, Russia). The surface topography was recorded in a semi-contact mode. A Nanovea T100 tribometer (Nanovea, Irvine, CA, USA) was used to perform the tests.
